# Uncertainty of chemical status in surface waters

**DOI:** 10.1038/s41598-021-93051-9

**Published:** 2021-07-01

**Authors:** Małgorzata Loga, Karol Przeździecki

**Affiliations:** Faculty of Building Services, Hydro and Environmental Engineering, Warsaw University of Technology, Nowowiejska 20, 00-653 Warsaw, Poland

**Keywords:** Environmental sciences, Engineering

## Abstract

This article addresses the issue of estimating P_om_—the probability of misclassifying the chemical status confidence of a water body status assessment. The main concerns of the authors were chemical quality elements with concentrations in water bodies which are close to or even smaller than the limit of quantification (LOQ). Their values must be set to half of this limit to calculate the mean value. This procedure leads to very low standard deviation values and unrealistic values of P_om_ for chemical indicators. In turn, this may lead to the false conclusion that not only is the chemical status *good* but also that this status assessment is perfect. Therefore, for a more reliable calculation of P_om_, the authors suggested a modified calculation in which the value of half the LOQ for calculating the mean value was kept, but zero as the concentration value for the standard deviation calculation was adopted. The proposed modification has been applied to the Hierarchical Approach procedure for P_om_ estimation of the chemical status of Polish rivers and lakes. The crucial finding is that current chemical status assessments may be incorrect in the case of approximately 25% of river water bodies and 30% of lake water bodies categorised as *good*, and 20% of both types of water bodies classified as *below good*.

## Introduction

The assessment of a water body status according to the Water Framework Directive (WFD)^1^ is a set of procedures in which water quality monitoring data gathered for all European water bodies are processed resulting in assigning to each water body (wb) its status class. The overall wb status is determined as the poorer one from its ecological and chemical status. The assessment of chemical status can only be twofold: *good* or *below good*. Whereas ecological status assessment is mostly based on five biological quality elements, chemical status assessment was originally based on 33 chemical substances, listed in Annex X to Directive 2000/60/EC^1^, called priority substances as they elimination from the environment is a priority. The detailed lists of priority substances and their threshold values separating *good* and *below good* statuses in the classification of water status were established in Directive 2008/105 /EC^2^. Both directives were amended by Directive 2013/39/EU^3^, where the list of priority substances was extended to 45 substances. Appropriate Environmental Quality Standards (EQS) were introduced for all these priority substances and some of them were selected as priority hazardous substances. The introduction of EQS aims to protect the most sensitive species from direct toxicity as well as humans via secondary poisoning. A smaller group of priority hazardous substances were identified as uPBT (ubiquitous, persistent, bioaccumulative and toxic)^4^ to which belong mercury, brominated diphenyl ethers (pBDE), tributyltin and certain polyaromatic hydrocarbons (PAHs).

Chemical status assessment is based on at least twelve measurements as stated in the Annex V of the WFD so it requires monthly measurements of each priority substance and other pollutants. Chemical status assessment is based not only on the annual average concentrations (AA-EQS) of the substances for which EQS are established, but also on the maxima expressed as maximum allowable concentration environmental quality standards (MAC-EQS). *Good* chemical status is defined as one in which no concentrations of priority substances in a wb exceed the relevant EQSs. As both threshold values: AA-EQS and MAC-EQS are set by Directive 2013/39/EU^3^, the chemical status assessment in all EU countries is performed in the same way.

The European Environmental Agency (EEA) in a water assessment report^5^ concluded that 40% of surface waters are in *good* ecological status or potential but only 38% are in *good* chemical status. This is why the ambitious goal of reaching *good* status by the year 2015 has been shifted to 2027 in order to give additional time to water managers to launch appropriate remediation actions.

Due to the fluctuation of river flows superimposed with random dynamics of point and non-point sources of pollution, concentrations of chemical pollutants measured over a period of one year can vary considerably^6,7^.The calculated annual mean and maximum values of concentrations from time series, treated as indices for chemical status assessment in the classification procedure, can lead to the overestimation or underestimation of the ‘true value’ of the index, or an incidental adoption of a result equal to the ‘true value’.

The resultant value of the indicator, i.e. the mean and/or maximum value as compared to legally set thresholds, can be responsible for misclassification of a wb, and may lead to essentially undesired consequences:

- false positive assessment (*good* status of a wb when its true status is *below good*). This can prevent implementation of corrective or remedial actions by water authorities;
- false negative assessment (*below good* status of a wb when the true status is *good*). This can trigger a decision of implementing unnecessary, difficult and costly remedial measures.

The details concerning estimation of probability in these two cases are presented in more detail in the “Data and methods” section.

Although it is defined in the WFD in a rather vague way, a certain measure of uncertainty has become a mandatory element of the reporting process.

The problem of status misclassification has been discussed in several papers^8–10^ but mostly in the context of ecological status. The literature includes numerous papers regarding the uncertainty of biological indicators concerning various water body types (rivers, lakes, reservoirs, transitional, etc.). Quantification issues related to errors in water sampling procedures, errors in chemical and biological analyses, estimates of uncertainty of water quality indicators, and river status assessment uncertainty have been addressed in many research reports and papers discussing monitoring of lakes and rivers^11–18^.

All these sources of uncertainty are inextricably linked to field and laboratory measurements. Errors concerning laboratory procedures^19,20^, however, are considered much smaller than those related to sampling in non-homogenous environments, transport of water samples, etc.^21^. From the point of view of status assessment, the standard deviation of the measured values plays an important role in the assessment of the mean value against thresholds defining classes.

In the case of considerable annual dynamics of pollutant concentrations, or bias of the mean value of a water quality indicator by the spatial heterogeneity of the waterbody (wb), accurate performance of sampling and further analytical measurements may still not result in an assessment of wb chemical status representing its actual chemical status.

Chemical status as it is introduced by WFD and the problems of its assessment have not been thoroughly discussed in literature^22,23^. Only recently have there been reports of mixtures of chemicals^24–26^ as well as studies focusing on diagnostic models of chemical pressures on ecological status^27,28^. In the first 15 years after the adoption of the WFD, most efforts have focused solely on ecological status^29^.

Quantification of the uncertainty of the ecological, chemical, and overall status of rivers was attempted^30^ but with relative precision applied as the measure of uncertainty instead of probability of misclassification. Regarding the chemical status assessment, it was concluded^30^ that assessments were highly uncertain as a result of logistic and economic conditions of the water monitoring system where the number of measured indicators of chemical status was low due to the cost-intensity of measurements.

Many water bodies show no occurrence of substances considered indicators of chemical status, as expected by the environmental legislation of the WFD^1^. If such substances do occur, they are often in very low concentrations. The values of monitored concentrations are frequently lower than the limit of quantification (LOQ), which is a concentration of the substance that can reasonably be determined with an acceptable level of accuracy and precision, or even lower than the detection limit of the method which is many times lower than the limit of quantification^31^.

The problem of dealing with data sets of concentrations below LOQ, i.e. censored data, particularly in the context of quality of environment, has been discussed by many authors^32–34^. The recently published, method^35^ is based on spatial modelling of concentrations close to LOQ but it cannot be applied to data, routinely collected within water monitoring. The adoption of a method involving arbitrary assigning of a numerical value to censored values leads to biased estimates of the mean value. At least three simple alternative options are available, namely the median, trimmed mean, and Winsorized mean^36^. With the exception of adoption of the median value instead of the mean value, the other methods consist of symmetrical changes on both sides of the data distribution values. For example, the trimmed mean is computed from a set of observations by eliminating the smallest *np* values (*np* is the number of values which are below LOQ) and the *np* largest values. Both methods are advised to be used when not more that 25% of the data are censored^37^.

Calculation of the mean value from a set of measured monitoring data with a share of censored data is regulated by Directive 2009/90/EC^31^, establishing technical specifications for the analysis and monitoring of chemical status of waters and it is further discussed in guidance^38^. Pursuant to the Directive 2009/90/EC^31^, measurement results which are below the LOQ should be set to half of the value of the relevant LOQ. The application of this rule increases the mean value from the measurement data when the substance is not actually present in the wb and its concentration is equal to zero, or when its true concentration is greater than zero but still below half the LOQ. On the other hand, such an approach decreases the true mean value when the actual concentration is below the LOQ but higher than half the limit. The Directive 2009/90/EC^31^ also provides a definition of ‘uncertainty of measurement’ as a parameter characterising the dispersion of quantity values attributed to a measurement. Unfortunately, it offers no suggestion on how to calculate the value of this uncertainty when data are censored.

It seems that the only attempt to present some estimations of confidence in assessing chemical status is available through the European Environmental Agency (EEA) web pages^4^. Unfortunately, the scale adopted to report the confidence of the chemical status assessment within the 2nd River Basin Management Plan by Member States is purely qualitative with three levels only. For Poland, 56% water bodies are characterized by low confidence, 30% by high confidence and the remaining 15% are unknown. The best picture appears for the Netherlands with 89% of water bodies with high confidence, 9% of medium, and 10% of unknown uncertainty. The highest percentage of water bodies with low confidence of chemical status was reported for Lithuania and Finland. Note, however, that there is a warning, added to these results pointing out that they are affected by the methods used in Member States and that they cannot be compared directly.

As for Poland, the method applied for estimation of confidence was based on the completeness of data only^39^. The highest level of confidence was assigned in case of chemical status assessment based on the full set of chemical indicators (at that time 33 substances) monitored at least 12 times per year. The lowest confidence was assigned when less than 50% of substances were monitored and the number of measurement data were lower than 10 in a year. Unfortunately, the description of the methods applied both to extrapolation and to uncertainty assessments for other countries were missing. There is also no information concerning the application of those confidence results for any practical water management issues.

This paper shows how to meet the obligation introduced by WFD and Guidance document No. 7^40^, i.e. how to estimate chemical status confidence defined as the probability that the indicator value estimated from the data points does in fact lie within some specified limits, or desired interval. The method presented in this paper of calculating probability of misclassification (P_om_) for chemical status fills the gap in reporting of chemical status uncertainty estimation. Together with estimated P_om_ for ecological status, it permits fulfilling the obligation of reporting the overall status assessment, together with the uncertainty measure, expected in the reporting procedure by the WFD. The study also focuses on the problem of dealing with censored data.

This paper presents a general overview of the bias existing in chemical status assessment. The sampling and chemical analysis procedures applied in chemical monitoring programmes carried out under Directive 2000/60/EC are validated and documented in accordance with EN ISO/IEC-17025^41^ standard or other equivalent standards accepted at international level^42^. Therefore, the presented results can be assumed representative of chemical status assessment not only in Poland, but also in other European countries with similar laboratory facilities.

## Data and methods

### Water quality monitoring data

This study is based on water quality monitoring data collected in the scope of the Polish National Monitoring System during 2016–2018, in both riverine and lacustrine water bodies throughout the territory of Poland. The number of monitored water bodies in particular categories and years is presented in Table 1.

**Table 1:** Number of water bodies monitored in the period 2016–2018.

Year	2016	2017	2018
No. of river wb	1302	1218	1715
No. of lake wb	199	206	266

The monitoring of chemical parameters included 45 chemical status indicators in accordance with the regulation concerning the classification used in the analysed period^43^.

Water bodies in Poland include 26 abiotic types of river waters, most of which are lowland rivers; rivers with organic substrate; and lowland sandy clay and gravel rivers^44^ as well as 13 types of lakes.

### Classification of chemical indicators

According to the regulation^43^, classifying a water body’s chemical status as *good* requires meeting both of the criteria tested with annual average (AA-EQS) and maximum allowable concentration (MAC-EQS) for all indicators. The exception is the group of pesticides: aldrin, dieldrin, endrin, and isodrin since the criterion for them is formulated as the threshold value for the sum of concentrations of these substances.

There are generally four cases of assigning classes to a chemical indicator:

(i) indicator is classified as *good* status class when both criteria for *good* status are met;
(ii) indicator is classified as *below good* status class when the mean concentration exceeds AA-EQS, whereas the maximum measured concentration is below the threshold;
(iii) indicator is classified as *below good* status class when the maximum concentration exceeds MAC-EQS, whereas the mean value from measurements is below the threshold for the mean; and
(iv) indicator is classified as *below good* status class when the annual mean and the maximum concentrations are above thresholds.

Any case of the assessment carries a possibility of misclassification, i.e. ascribing a class different than the actual class.

As in the works by Clarke and Herring^8^ and Kelly^45^, also in this paper, P_om_ is understood as the probability of committing a type II error, i.e. accepting the null hypothesis H_0_ consisting of accepting a *below good* class when the true class is *good* or vice versa.

### Probability of misclassification calculation method

Due to the difficulty of determining the actual value of the index (i.e. representing the true status of a wb) and thus to which class a water body status does belong, the class indicated by the average value of measurement data for the chemical quality element, at a given measurement location within a certain period of time, is considered as the resulting status assessment. This quantity, commonly used in the monitoring of surface waters to indicate the class is, however, a random variable—the random realization of a variable of which does not necessarily indicate the class to which the estimated true value belongs.

When only the criterion based on annual average is considered, P_om_ can be calculated from the following formula:

1 $${P}_{om}=1-\underset{l(i)}{\overset{u(i)}{\int }}g\left(\stackrel{-}{x}\right)dx$$

where:

*l*(i) , *u*(i) are the specified lower and upper limits of the true class ‘i’ of the indicator mean value. $g\left(\stackrel{-}{\mathrm{x}}\right)$ is the distribution function for the indicator mean value.

This study assumes that the distribution function g $\left(\stackrel{-}{\mathrm{x}}\right)$ of the average value of the chemical element $\stackrel{-}{\mathrm{x}}$ can be approximated by the Student's t-distribution function, where two parameters of this distribution—the expected value E(x) and the standard deviation σ(x), result directly from the statistical assumptions: E $\left(\stackrel{-}{\mathrm{x}}\right)$ = E (x) and σ $\left(\stackrel{-}{\mathrm{x}}\right)$ = σ(x)/√n, where n is the numerical size of a finite sample of measurements of the chemical element x. This means that, in practice, the values of the statistical parameters of the distribution g $\left(\stackrel{-}{\mathrm{x}}\right)$ are calculated directly from the values of statistical parameters of the Student's t-distribution estimated on the basis of the measurement data of the chemical element x. Developing this method, a conscious assumption was made that the measurement bias was negligible, as biases values were simply missing from the whole data base. It was noted that this assumption may have an effect on the results, but taking into account that measurements which results were stored in the data base were performed by many people, in three different years, using different devices with different precision, it was concluded that this approach was the only reasonable one.

The P_om_ value for an indicator depends on the distance between the mean or maximum value and the boundary of the class, and on the standard deviation characterising the width of measurement distribution.

Due to the heterogeneity of the water environment in both horizontal and vertical directions, temporal evolution of biochemical processes, and temporal variability of pollution discharges, mean values of concentrations of dissolved substances frequently show considerable variance^46–48^.

In the presented methodology authors have not taken into account the impact of possible correlations between chemical parameters on P_om_ value. Correlation among chemical indicators might be significant and could be dealt with by using for example PCA as shown in^49^. Unfortunately in case of existing data base of monitoring results, there was a different number of measured parameters for different water bodies and all the sample sizes were relatively small. Including the possible effect of correlation between parameters, would make the task of uncertainty assessment very challenging. It is very likely that the whole procedure would complicate the calculations to the point when they cease to be applicable and thus useful for wider community consisting not only from researchers but also water managers and water administration.

The calculation of P_om_, taking into consideration the criterion of maximum concentration, employed the right-hand side Gumbel distribution type I. For both Student's t-distribution and Gumbel distribution, functions from the Python package Scipy^50^ were applied to fit the distribution models.

In line with the definition given above, the probability of misclassification (P_om_) is the probability of committing the type II error. The H_0_ hypothesis is defined in agreement with the result of the chemical class accessed either on the basis of the mean value or on the maximum value. In case of false positive assessment of the class, P_om_ means the probability of the rejection hypothesis H_0_ stating ‘chemical status class is *below good*’ while the true class is *below good*. On the other hand in case of false negative assessment, P_om_ expresses the probability of the rejection of hypothesis H_0_ stating ‘chemical status class is *good*’ while it is correct. It means that, for each indicator, P_om_ means the probability that the class is assessed incorrectly. A similar situation occurs in the case of the calculation of the probability of misclassification for the maximum value, i.e., either the *good* status class is determined when *below good* is the actual class, or vice versa.

The entire data processing was performed in Python 3 using the Numpy^51^, Scipy^50^, Pandas^52^ libraries for analysis purposes and the Matplotlib^53^ and Seaborn libraries^54^ for visualization purposes.

### Hierarchical approach of chemical status assessment

The assessment of chemical status misclassification requires taking into consideration uncertainties in all chemical indicators. Following the Hierarchical Approach introduced by Loga^9^, but mainly for ecological status^55^ the Hierarchical Approach presented in this paper was tailored to estimate the uncertainty of chemical status assessment. In this study, the assessment of chemical status was based on monitoring data separately for each year. When all chemical indicators were not available (not all substances included in the chemical status assessment procedure were measured), the chemical status was assessed from the available chemical indicator values by applying One-Out-All-Out rule (OOAO) and then its uncertainty measure with the aid of the Hierarchical Approach.

The general idea of the Hierarchical Approach is to adopt a procedure similar to that in the OOAO rule, where the indicator classified in the worst class is the decisive indicator as it determines the result of classification. Unlike for ecological status assessment where there are five classes, for chemical status there are no intermediate classes between *good* and *below good* so in case of just one chemical indicator classified in the *below good* class the chemical status of water body is downgraded to the *below good*. Taking the same approach for the chemical status uncertainty assessment, the probability of misclassification of the indicator decisive for the chemical status is assumed to be the probability of misclassification of this status.

Because chemical status assessment is based on two criteria, namely, annual mean and annual maximum value, errors are possible for both criteria independently. Therefore, two P_om_ values are assessed for each chemical indicator.

**Figure 1:**
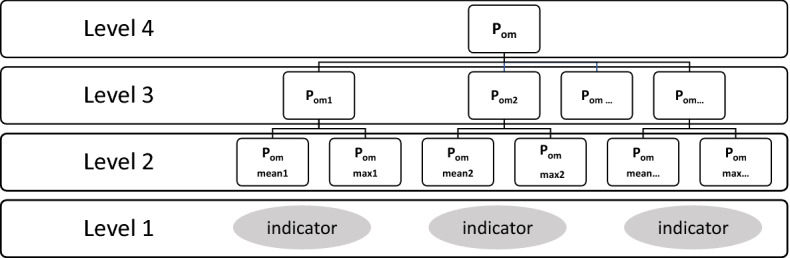
Hierarchical Approach for assessing Probability of Misclassification for the chemical status for a water body. Level 1 consists of classified chemical quality indicators (*good* or *below good* status class); Level 2 consists of P_om_s for the class of each indicator; there are separate P_om_s for the mean and max criterion; Level 3 represents eventual P_om_ values for all chemical indicators; and Level 4 is the final P_om_ for chemical status.

The modified version of the Hierarchical Approach for assessing misclassification of chemical status is presented in Fig. 1. It has to be noted that **t**here are differences in following the hierarchical process for water bodies classified in *good* and *below good* chemical status.

When water bodies are classified as *good* chemical status, the lowest level of hierarchy (Level 1) depicted in Fig. 1 represents all indicators classified as *good* chemical class, as both criteria of good chemical status are fulfilled. For every indicator on the second level (Level 2), both probabilities are calculated: for the mean value criterion P_om mean_ and maximum value criterion P_om max_. On the third level (Level 3) of hierarchy, P_om_ values for each indicator are assigned by choosing the larger of the two values calculated on the lower level. On the highest level of hierarchy—Level 4, the largest value of P_om_ for indicators is chosen as the final P_om_ of the chemical status for a given water body.

In the case of a water body assessed as *below good* chemical status, at least one of its indicators of chemical status was ascribed to the class *below good*. In such a case, only indicators ascribed to this class are taken into account on the first level of the hierarchy (Level 1), because indicators assessed as *good* are not decisive for the status class.

The second level (Level 2) consists of P_om_ values for mean and maximum values of indicators classified in *below good* class.

The third level of hierarchy (Level 3) is the most complex one and it depends on the reason why indicators were classified to the *below good* class (one of three cases listed earlier (ii)–(iv) in “Classification of chemical indicators” in “Data and methods” section).

In case (ii) and (iii) when an indicator is classified in the *below good* status class i.e. when the mean concentration exceeds AA-EQS or maximum concentration exceeds MAC-EQS, the P_om_ for this indicator takes the value of P_om_ estimated for the criterion decisive for the *below good* status (mean ii) or maximum iii)).

The fourth possibility (iv) applies when both annual mean and maximum values are higher than the corresponding AA -EQS and MAC-EQS. Then, the probability of inaccurate class assessment involves committing two errors simultaneously, i.e. inaccurate classification of both the annual mean and the maximum. Therefore, the P_om_ for this chemical indicator is calculated as a product of the two corresponding P_om_s.

The third level of hierarchy (Level 3) consist of P_om_ values for all chemical indicators used for chemical status assessment.

At the Level 4 of the Hierarchical Approach the highest value among Level 3 P_om_’s is adopted as the P_om_ for chemical status.

The description above corresponds to the uncertainty assessment for classification of every substance included in the chemical status assessment procedure.

### Modification of standard deviation calculation

Because chemical status is based on substances which either are not present in water bodies or occur at very low concentrations, the measured values are frequently very close to the LOQ or even the detection limits.

When all the measured concentrations are smaller than the LOQ, and are assigned half of this limit (EC, 2009/90/EC), the standard deviation from the set of identical values is equal to zero. From the statistical point of view, the zero value of standard deviation suggests the most efficient estimator of the expected value. It gives a false impression that the class assessed based on this indicator value (the mean value of the measured concentration) was assessed with high precision. The more measurement results are below the limit of quantification, the smaller the value of the standard deviation becomes, resulting in a decrease in uncertainty whereas it is not true .The true value of the measurement reported as ‘below limit of quantification’ can be any value from zero (including zero) to the value of the LOQ. Therefore, the conventional standard deviation formulae lead to wrong conclusions when applied to a series of measurements below the LOQ.

To find a way out of this erroneous approach which distorts the estimation of confidence in the wb status, particularly when the *good* chemical status was adopted with relatively high values of detection limits in laboratory chemical procedures, it is useful to introduce a modified approach for calculating the standard deviation. When using the definition formula for calculating the standard deviation whenever the real measurement (x_i_) value is below the limit of quantification, it is beneficial to set the value to zero (instead of half of the LOQ) but keep the value of half the LOQ for calculating the mean value. Therefore a modified standard deviation proposed in this approach can be calculated from the following formulas:

2 $$mod\,\,stdev\,\,=\,\,\sqrt{\frac{1}{N}\left[{({x}_{1}-\stackrel{-}{x})}^{2}+{\dots +({x}_{i}-\stackrel{-}{x})}^{2}+\dots\,\,+{({x}_{N}-\stackrel{-}{x})}^{2}\right]}$$

and

$${\stackrel{-}{x}-mean\,\,value\,\,;\,\,\,\,\,\,\,\,\,\,\,\,x}_{i}=\left\{\begin{array}{c}0\,\,for\,\,{x}_{i}<LOQ\\{x}_{i}\,\,for\,\,{x}_{i}\ge\,LOQ\end{array}\right.{i}=\quad{1,2}\ldots N$$

Taking the value zero instead of half the limit of quantification results in a bigger standard deviation value when measurement results are below the LOQ. In fact, the same result could have been reached using the LOQ value instead of zero as, in both cases, the distance from LOQ/2 was maximized.

## Results

The study results are divided into three subchapters in order to emphasise three evident outcomes: the results of introducing a modified way of taking into account censored data in the assessment of uncertainty; the results of the uncertainty analysis of particular chemical indicators, i.e. priority substances; and the results of the uncertainty of chemical status based on the example of water bodies of Polish rivers and lakes.

### Comparison of uncertainty for the conventional and modified approach to taking into account censored data for calculation of standard deviation

The differences of application to the assessment of P_om_ of the standard deviation calculated for below the LOQ concentrations with the conventional formula, versus the modified approach are shown in Table 2.

**Table 2:** Comparison of results of the probability of misclassification evaluation for chemical indicators with the application of conventional standard deviation with the suggested modified formula for calculating standard deviation for measurements including censored values.

Examples of data from three lacustrine water (LW) bodies for three chemical indicators	(a) Benzo(a)pyrene		(b) Fluoranthene		(c) Endosulfan	
	PLLW10035		PLLW10051		PLLW10015	
	Raw data (µg/l)	Data for calculation (µg/l)	Raw data (µg/l)	Data for calculation (µg/l)	Raw data (µg/l)	Data for calculation (µg/l)
	< 0.00005 0.0008 0.00008 < 0.00005 < 0.00005 < 0.00005	0.000025 0.0008 0.00008 0.000025 0.000025 0.000025	0.009 0.003 < 0.001 < 0.001 0.007 < 0.001	0.009 0.003 0.0005 0.0005 0.007 0.0005	< 0.001 < 0.001 < 0.001 < 0.001 < 0.001 < 0.001	0.0005 0.0005 0.0005 0.0005 0.0005 0.0005
AA-EQS	1.7 × 10^–4^		0.006		0.005	
Sample mean value	1.6 × 10^–4^		0.0034		0.0005	
MAC-EQS	0.27		0.12		0.01	
Sample max. value	0.0008		0.009		0.0005	
Class assessed	*Good* class		*Good* class		*Good* class	

	Conv std. dev	Modif std. dev	Conv std. dev	Modif. std. dev	Conv. std. dev	Modif. std. dev
St. dev	0.00031	**0.00032**	0.0037	0.004	0	0.00055
P-probability of *good* class (based on mean)	0.2	**0.19**	0.5	**0.53**	1	**0.80**
P_om_ probability of misclassification (based on mean)	0.80	**0.81**	0.44	**0.47**	0	**0.20**
P probability of *good* class (based on max)*	0.82	**0.84**	0.92	**0.82**	1	**0.53**
P_om_ probability of misclassification (based on max)*	0.18	**0.16**	0.08	**0.18**	0	**0.47**
P_om_ (level 3)	0.80	**0.81**	0.44	**0.47**	0	**0.47**

Values calculated by means of the modified formula are in bold.

*P probability of *good* class, *P_*om*_ probability of misclassification i.e. probability of *below good* class.

These differences are relevant for Level 2 of the Hierarchical Approach (Fig. 1). Three examples of data are provided in the upper part of Table 2, i.e. censored concentrations of three chemical substances in different lacustrine water bodies and data transformed for the purpose of calculation of the mean value. Standard deviations and probabilities of the class (P), together with probabilities of misclassification (P_om_) calculated in line with the conventional (Excel) formulas are contrasted with the P and P_om_ resulting from the modified formula for st. dev for censored data.

A detailed calculation of the modified standard deviation for benzo(a)pyrene, as in the Table 2—example (a) is as follows:

$$mod\,\,stdev=\sqrt{\frac{1}{5}\sum \left[{(0-0.00016)}^{2}+{(0.0008-0.00016)}^{2}{+(0.00008-0.00016)}^{2}{+(0-0.00016)}^{2}+{(0-0.00016)}^{2}{+(0-0.00016)}^{2}\right]}=0.000322$$

In the example (a) of Table 2, only two data points of benzo(a)pyrene are greater than the LOQ. The *good* class is assigned to this indicator because neither the AA-EQS nor MAC-EQS value was exceeded. The P_om_ of the *good* class for benzo(a)pyrene when calculated by means of the conventional method is a high value, i.e. 0.80 but with the standard deviation calculated in the modified way it is slightly higher. For fluoranthene, the corresponding P_om_ values are generally lower than in case (a), but higher than those based on the conventional st. dev. formula, and still almost reaching the value of 0.5.

A comparison of P_om_ values for endosulfan is hardly possible because the zero value of standard deviation does not permit reporting any uncertainty related to the classification for this indicator. This is clearly not acceptable as there is always uncertainty in class assessment for any indicator.

The uncertainty of chemical status based on P_om_ as a measure of this uncertainty was assessed for all the analysed riverine and lacustrine wbs. For better presentation of the results, the range of P_om_ values was divided arbitrarily into four intervals, namely < 0, 0.1 > , (0.1, 0.3 > , (0.3, 0.5 > , and (0.5, 1 > .

**Figure 2:**
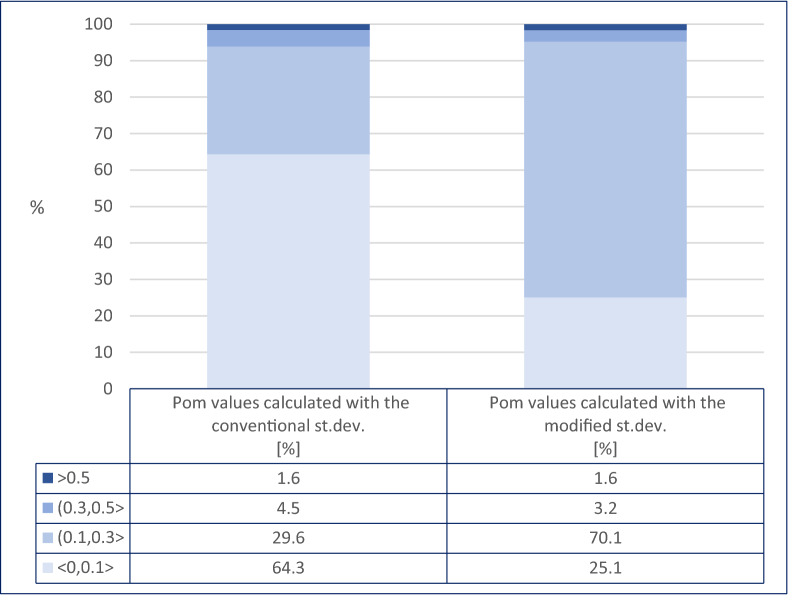
Comparison of P_om_ values calculated with the conventional (stacked bar plot on the left) and modified (stacked bar plot on the right) st. dev in the case of measurements below the LOQ. The comparison is based on all chemical indicators in riverine water bodies classified in the *good* class in 2017. The range of P_om_ values is divided into four intervals: < 0, 0.1 > , (0.1, 0.3 > , (0.3, 0.5 > , and (0.5, 1 > . For each interval, the percentage of indicators with P_om_ belonging to this interval is presented.

The comparison of P_om_s for chemical indicators, i.e. the results from the third level of hierarchy (Fig. 1) assessed based on the conventional formula for standard deviation with P_om_ values based on the modified formula, shows no differences for indicators classified in the *below good* class However, there are significant differences in percentages in the P_om_ intervals, between results reached with conventional and modified formulas, for indicators assessed in the *good* class. These differences in distribution among four intervals of P_om_s are presented in Fig. 2 based on the example of wbs in 2017, representative both for river and lake waterbodies over three years.

The bar plots in Fig. 2 evidently show that the greatest difference in abundance occurs between the percentage of indicators belonging to the first two intervals of P_om_ s, i.e. < 0, 0.1 > and (0.1, 0.3 > . The interval characterised by the lowest P_om_ decreased two and a half times when the modified st. dev was applied to P_om_ estimation, whereas the group with P_om_ values within a range of (0.1, 0.3 > increased in almost the same proportion. The percentage of the fourth interval, i.e. the highest P_om_, was unaffected by the change in the formula of standard deviation. It should be emphasised that the modified way of calculating standard deviation is only important for estimating P_om_ for chemical indicators characterised by very low concentrations. On the other hand, it permits improved performing of the complete assessment of chemical status uncertainty.

### Probability of misclassification for chemical indicators for Polish riverine and lacustrine water bodies

**Figure 3:**
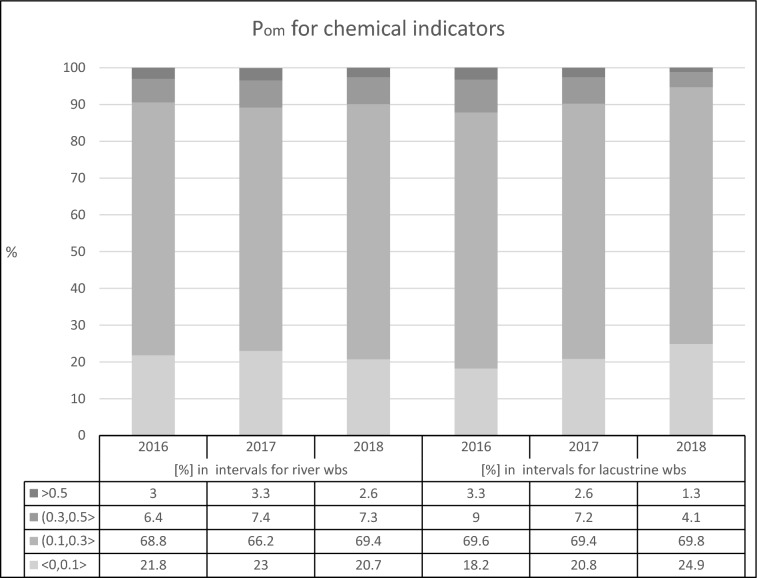
Probability of misclassification (P_om_ ) of all chemical indicators (Level 3) for riverine and lacustrine water bodies in the years 2016–2018. The range of P_om_ values is divided into four intervals: < 0, 0.1 > , (0.1, 0.3 > , (0.3, 0.5 > , and (0.5, 1 > . For each interval, the percentage of indicators with P_om_ belonging to this interval is presented.

The modified formula for calculating the standard deviation for censored data and P_om_ assessment was applied to all chemical indicators and to all riverine and lacustrine water bodies. The results of this assessment, corresponding to Level 3 of the hierarchy (Fig. 1), are presented in Fig. 3.

The monitored riverine water bodies are several times as numerous as the lacustrine ones (Table 1). Nonetheless, a striking similarity is observed in the percentage of chemical indicators belonging to each of the four intervals, defined by the range of the probability of misclassification presented in Fig. 3. The group characterised by P_om_ greater than 0.5 contains no more than 3.5% of indicators. The interval characterised by the smallest values of P_om_ consist of 20–25% of the indicators of chemical status. The greatest number of indicators is characterised by P_om_ within a range of (0.3,0.5 >.

### Probability of misclassification of chemical status

Results of the probability of misclassification of the chemical status of water bodies, i.e. P_om_ values corresponding to Level 4 of the hierarchy, are presented in Table 3. The results for all years are lumped together but presented separately for *good* and *below good* status. The two main columns on the left of Table 3 display values of P_om_ resulting from conventional and modified approach to st. dev. for censored data. Chemical substances, on the basis of which *below good* status was assessed, occurred in waters in concentrations much higher than LOQ. This is the reason that assessment of P_om_ values for wbs in *below good* chemical status was not affected by the method of calculation of st. dev. and that is why there are single columns for both types of wbs in below good status in Table 3. Similarly to the way of presenting P_om_ for indicators, P_om_ values for chemical status were discretized into four intervals depending on the P_om_ value.

**Table 3:** Probability of misclassification of chemical status (level 4) for riverine and lacustrine water bodies for the years 2016–2018. Comparison of the probability of misclassification based on conventional and modified std. dev*).

	% of chemical status with four intervals of P_om_ for riverine wb in *good* status		% of chemical status with four intervals of P_om_ for lacustrine wb in *good* status		% of chemical status with four intervals of P_om_ in *below good* status	
P_om_ range	Conventional st. dev	Modified st. dev	Conventional st. dev	Modified st. dev	Riverine wb	Lacustrine wb
< 0, 0.1 > (0.1, 0.3 > (0.3, 0.5 > (0.5, 1. >	28.5 29.3 17.2 25	11.7 30.4 32.8 25.1	23.5 27.9 18.1 30.4	10.3 30.4 28.9 30.4	13.1 37 31.5 18.4	0.9 16.8 62.3 20

*P_om_s for wbs in *below good* chemical status uncertainty of the assessment was not affected by the method of calculation of st. dev.

There are differences in percentage in the first three intervals in the columns for *good* chemical status but the values for the fourth interval i.e. (0.5, 1 > are unchanged. For both types of wbs, this comparison of intervals for P_om_ shows a clear decrease in the percentage within the lowest value of P_om_ and an increased percentage of P_om_ values belonging to the (0.3, 0.5 > interval. Changes within the (0.1, 0.3 > interval are rather insignificant. The overall results of uncertainty of *good* chemical status for lakes and rivers when the modified st. dev. was applied are more pessimistic than that of the conventional approach, as only around 12% of wbs have a certain *good* status. Intervals of P_om_ values for chemical status smaller than 0.1 are considerably more numerous for *good* status than for *below good*.

From the above table (Table 3) it can be concluded that, for about 40% of riverine and lacustrine water bodies, their *good* chemical status was assessed with a probability of misclassification below 0.3. The most difficult to accept, especially for water managers, is that an incorrect assessment of status has been made in approximately 25–30% of lake and river water bodies previously assigned the *good* chemical status and about 20% of those with *below good* status. According to the authors, this is the interpretation of the probability of misclassification higher than 0.5.

## Discussion

There are many publications and reports criticizing the whole concept of chemical status assessments as introduced by WFD^1^ and, through this failure, the attempts of status remediation however apart from the draft report EEA^5^ and web EEA pages^4^, the issue of the uncertainty of the assessed chemical status does not appear in any document relating to water management.

There was an earlier attempt to apply the hierarchical approach to the chemical status of water bodies in three voivodships in Poland^55^, but due to the scarcity of measurement data of many priority substances and thus not representative character of assessments, the results were mostly focus on ecological status. The additional constraint encountered was the problem with dealing with left-side censored values of chemicals concentration. It was difficult to accept zero value of Pom as shown on the example of endosulfane in Table 2.

When assessing chemical status of a water body as *below good,* it is not reported to the European Commission how many and which substances for wbs categorized *below good* violated the threshold values, nor how big the discrepancies were^56^.The same authors suggest that the chemical status assessment is also ill-defined due to excluding many substances from the list obligatory for chemical monitoring. They suggest that if the list of chemicals is enlarged, there would be no water body in the EU territory in *good* chemical status. However, now with 45 priority substances, as many as 62% of wb have been classified as failing to reach good chemical status^5^. There was a First Watch List for pollutants including emerging ones initiated in 2015^57^ for which the available monitoring information was considered as insufficient.

It has been concluded that EQS established as set by Directive 2008/105/EC^2^ aims to protect the most sensitive species from direct toxicity and humans via secondary poisoning^5^ but are not significantly protective against mixture effects. Moreover, there is an urgent need to revise the tools used to assess the safety of chemical pollution^24,58^.

The most important problems, together with recommendations aiming at reaching effective methods of assessments and management concerning chemical pollutants, were presented by Brack^59^. These are the conclusions of the NORMAN network^60^ and FP7 Collaborative Project SOLUTION^61^. There are altogether ten main problems grouped around three basic recommendations as “improve monitoring, and strengthen comprehensive prioritization, foster consistent assessment and support solution-oriented management”. The issue of uncertainty of chemical status assessment presented in this paper is strictly connected with the first recommendation put forward by Brack^59^ i.e. modification and improvement of the monitoring system. The results of measurements below LOQ of substances such as tributyltin, benzopyrene, benzofluoranthene or endosulfan as well as many others, caused by their hydrophobic nature resulting in very low solubility in water, can be overcome by introduction of passive sampling methods and biota monitoring to monitoring programmes instead of their measurements in water^42,62^.Since a high uncertainty of biological indices may be found for measurements performed just once in the six-year River Basin Management Plan, it can be expected that measurement values of substances accumulated in the biomass, if performed also only once per the six-year period, similarly will result in high uncertainty in the assessment of the chemical status. However, before these new methods will be operational, the currently run monitoring gives results which are highly uncertain. It is also important to notice that, for this passive and biota monitoring, there is also a necessity to prepare an uncertainty assessment method. The Hierarchical Approach presented here seems feasible for this purpose. This kind of approach has been already put into practice in assessing water status uncertainty for ecological, chemical and overall status of water bodies in Poland by water administration.

Concerning changes which are required in future water quality monitoring in order to upgrade protection from chemical mixtures risks and improve management of water bodies that are at greatest risk, several sound recommendations are formulated as results of European Collaborative Project SOLUTIONS^63,64^. These are inclusion of mixture effects of substances below experimental no observed adverse levels, extend the impact assessment of multiple receptors, replace traditional surveys, by effect-based monitoring and assessment methods. Definitely these recommendations cannot be introduced at short notice and without changes in legislation. Therefore, it is necessary to amend the WFD and build feedback links between the WFD and other EU legislation on industrial chemicals, pesticides, biocides and pharmaceuticals^63^.

As for the needs expressed occasionally by water managers concerning monitoring, these are typically requests for cost-efficient methods of monitoring future threats to water quality^38,59^, not demands for narrower uncertainty intervals for water status. It seems that the more appropriate approach to improve existing monitoring programs would be to ask water managers about the satisfactory confidence from their point of view and, according to the accepted width of this uncertainty interval, the necessary frequency of sampling and methods of monitoring can be prepared.

## Conclusions

Adopting the value of half the LOQ in the case of left-side censored concentrations of substances can increase the mean value in comparison to the case where data points below LOQ are set as zero, when the true concentration is higher than half the LOQ but still below it. It can decrease the mean value in the opposite situation. When chemical quality elements are classified in the *good* class, more measurement results below the limit of quantification show a smaller value of the standard deviation. It is in fact the reverse, because the true measurement value can be any value between zero (including zero) and the limit of quantification. The proposed method of calculating the standard deviation in the case of censored data improves the results of the estimation of uncertainty for chemical indicators. It also permits reliable and complete assessment of chemical status uncertainty.

The Hierarchical Approach to the assessment of probability of misclassification of chemical status applied in this study fills the gap in reporting the chemical status together with its uncertainty measure.

Less than half of all water bodies in Poland monitored in the period 2016–2018 assessed in *good* chemical status are characterised by relatively low (lower than 0.3) probability of misclassification. Therefore, their *good* chemical status is rather certain and no remedial measures should be undertaken within water management plans for these water bodies. The most crucial finding is that current chemical status assessments may be incorrect in the case of approximately 25% of river water bodies and 30% of lake water bodies categorised as *good*, and 20% of both types of water bodies classified as *below good*.

It is very likely that in countries such as Poland, where the obligation to test an increasing number of substances included in the EQS list and to extend monitoring onto biota if operational, will lead to attempts to economize on the monitoring programs, which is already the case for the ecological status. A principle of inheritance of the assessment from previous years currently operates for those indicators whose measurements are not performed in the current monitoring cycle. This means that the assessments of chemical indicators and their uncertainties obtained from the previous monitoring periods can be used for some time. That is why the uncertainty issues of chemical status presented in this paper should be known to water managers, which it is not the case at present.

Unfortunately, the question whether the presented range of status uncertainty is satisfactory to water managers remains open. Due to the high cost of measurements of priority substances and other hazardous substances, another question arises: Is there any possibility to decrease the sampling frequency, inevitably leading to an increase in uncertainty?
